# Blockade of Cyclophilin D Attenuates Oxidative Stress-Induced Cell Death in Human Dental Pulp Cells

**DOI:** 10.1155/2019/1729013

**Published:** 2019-04-04

**Authors:** Shengbin Huang, Bingbing Zheng, Xing Jin, Qihao Yu, Xiaorong Zhang, Xiaoyu Sun, Yuting Chen, Xuerui Ren, Daniel Wismeijer, Jianfeng Ma, Chengfei Zhang, Gang Wu, Yihuai Pan

**Affiliations:** ^1^Department of Prosthodontics, School and Hospital of Stomatology, Wenzhou Medical University, Wenzhou, China; ^2^Institute of Stomatology, School and Hospital of Stomatology, Wenzhou Medical University, Wenzhou, China; ^3^Department of Oral Implantology and Prosthetic Dentistry, Academic Centre for Dentistry Amsterdam (ACTA), University of Amsterdam and Vrije Universiteit Amsterdam, 1081 LA Amsterdam, Netherlands; ^4^Department of Endodontics, School and Hospital of Stomatology, Wenzhou Medical University, Wenzhou, China; ^5^Comprehensive Dental Care, Endodontics, Faculty of Dentistry, The University of Hong Kong, Pokfulam, Hong Kong

## Abstract

Pathological stimuli, such as bacterial activity, dental bleaching, and nonpolymerized resin monomers, can cause death of dental pulp cells (DPCs) through oxidative stress- (OS-) induced mitochondrial dysfunction. However, the crucial molecular mechanisms that mediate such a phenomenon remain largely unknown. OS is characterized by the overproduction of reactive oxygen species (ROS), e.g., H_2_O_2_, O_2_^−^, and ^·^OH. Mitochondria are a major source of ROS and the principal attack target of ROS. Cyclophilin D (CypD), as the only crucial protein for mitochondrial permeability transition pore (mPTP) induction, facilitates the opening of mPTP and causes mitochondrial dysfunction, leading to cell death. In the present study, we hypothesized that CypD-mediated mitochondrial molecular pathways were closely involved in the process of OS-induced death of human DPCs (HDPCs). We tested the phenotypic and molecular changes of HDPCs in a well-established OS model—H_2_O_2_ treatment. We showed that H_2_O_2_ dramatically reduced the viability and increased the death of HDPCs in a time- and dose-dependent manner by performing MTT, flow cytometry, and TUNEL assays and quantifying the expression changes of Bax and Bcl-2 proteins. H_2_O_2_ also induced mitochondrial dysfunction, as reflected by the increased mitochondrial ROS, reduced ATP production, and activation of mPTP (decreased mitochondrial membrane potential and enhanced intracellular Ca^2+^ level). An antioxidant (N-acetyl-L-cysteine) effectively preserved mitochondrial function and significantly attenuated H_2_O_2_-induced cytotoxicity and death. Moreover, H_2_O_2_ treatment markedly upregulated the CypD protein level in HDPCs. Notably, genetic or pharmacological blockade of CypD significantly attenuated H_2_O_2_-induced mitochondrial dysfunction and cell death. These findings provided novel insights into the role of a CypD-dependent mitochondrial pathway in the H_2_O_2_-induced death in HDPCs, indicating that CypD may be a potential therapeutic target to prevent OS-mediated injury in dental pulp.

## 1. Introduction

Dental pulp is highly vulnerable to various physicochemical and microbiological stimuli, such as acute injury, bacterial activity and metabolites, dental bleaching, and nonpolymerized resin monomers [1–3]. These stimuli can cause dental pulp cell (DPC) death, eventually leading to irreversible pulp inflammation and necrosis [1]. One main pathogenic mechanism of these stimuli is oxidative stress- (OS-) mediated damage in DPCs [4, 5]. OS is characterized by the excessive generation of reactive oxygen species (ROS), e.g., H_2_O_2_, O_2_^−^, and ^·^OH [6]. OS stimulated by dental composites and lipopolysaccharides has been reported to induce cell cycle alteration and death of human DPCs (HDPCs) [7]. During dental bleaching, exogenous hydrogen peroxide (H_2_O_2_) released from the bleaching solution can result in the cell death of DPCs [5].

Mitochondria are the main resource and also the major attacking target of ROS [8]. In response to the abovementioned pathological stimuli, the excessively generated ROS will cause rapid depletion of antioxidants and then induce oxidative damage to mitochondria, which subsequently lead to mitochondrial dysfunction in HDPCs [9]. Such a cell death can be attenuated by the application of mitochondria-specific antioxidants [10]. These results indicate that mitochondrial dysfunction is highly involved in OS-induced HDPC death. However, the underlying molecular mechanisms remain largely unknown.

The mitochondrial permeability transition pore (mPTP), assembled between the inner and outer mitochondrial membranes, opens with the relatively severe disturbance of intracellular redox and/or Ca^2+^ homeostasis [11, 12]. The mPTP opening can lead to a solute exchange between mitochondrial matrix contents and the surrounding cytoplasm, which is commonly linked to mitochondrial dysfunction [13]. Cyclophilin D (CypD) is a critical protein for mPTP opening [14]. CypD-dependent mPTP opening has been shown to play a key role in ROS-induced mitochondrial dysfunction [15] and cell death [14]. The pharmacological inhibition or genetic ablation of CypD can rescue mitochondrial dysfunction and cell damage induced by OS [16]. However, it remains unknown whether the CypD-dependent mitochondrial pathway is involved in the OS-mediated death of HDPCs. In this study, we aimed to identify the potential role of CypD in the regulation of mPTP and mitochondrial dysfunction in the OS-induced HDPC death.

## 2. Materials and Methods

### 2.1. Informed Consent and Ethical Approval

The human research commission of the School and Hospital of Stomatology, Wenzhou Medical University, Wenzhou, China (2018001), gave approval to the study. For the human tooth collection, a written informed consent was obtained from all the subjects.

### 2.2. Reagents

Cell culture medium and additional supplements were bought from Life Technologies (Grand Island, NY, USA). The antibodies were obtained from Cell Signaling Technology (Beverly, MA, USA). The chamber slides and 4′,6-diamidino-2-phenylindole (DAPI) were from Invitrogen (Carlsbad, CA, USA). MitoSOX Red, TMRM, and MitoTracker Green (MTGreen, Molecular Probes) were from Life Technologies (Grand Island, NY, USA). A terminal deoxynucleotidyl transferase deoxyuridine triphosphate nick-end labeling (TUNEL) kit was from Roche (Mannheim, BW, Germany). 3-(4,5-Dimethylthiazol-2-yl)-2,5-diphenyltetrazolium bromide (MTT), an annexin V-fluorescein isothiocyanate (FITC) detection kit, H_2_O_2_, and N-acetylcysteine (NAC) were from Sigma-Aldrich (St. Louis, MO, USA). Cyclosporine A (CsA) was from Cell Signaling Technology (Beverly, MA, USA). An adenosine triphosphate (ATP) assay kit was from Beyotime Institute of Biotechnology (Shanghai, China).

### 2.3. Cell Culture, Characterization, and Treatment

HDPCs were isolated from the dental pulp tissues of noncarious third molars and grown in Dulbecco's modified Eagle's medium (DMEM) containing 10% fetal bovine serum (FBS), 100 U/mL penicillin, and 100 mg/mL streptomycin (Gibco, Grand Island, NY, USA). Cultures were maintained in a humidified atmosphere containing 5% CO_2_ at 37°C. For HDPC lineage characterization, the morphological analysis was performed, and the cells were immunocytochemically stained for vimentin and keratin. The working concentrations of the compounds were as follows: NAC (2.5 mM) and CsA (2 *μ*M). The final concentration of dimethyl sulfoxide (DMSO) in the culture was less than 0.5% in all the experiments. The cells with or without H_2_O_2_ were treated with the indicated test compounds according to the experimental protocol.

### 2.4. Cell Viability

HDPCs were seeded in 96-well plates and treated with the indicated reagents. Then, HDPCs were incubated with 10 *μ*L MTT solution (5 mg/mL). After 4 h, the formazan crystals were dissolved by DMSO. The plates were then measured in a microplate reader. The optical density (OD) of the control group in MTT was taken as 100% viable.

### 2.5. siRNA-Mediated CypD Knockdown in HDPCs

CypD siRNA targeting human peptidylprolyl isomerase F (PPIF) and the negative control (NC) were purchased from RiboBio (Guangdong, China) and transfected in HDPCs with Lipofectamine 3000 (Invitrogen, Carlsbad, CA, USA) according to the manufacturer's protocol.

### 2.6. Measurement of Cell Death by Flow Cytometry and TUNEL Assays

The cell death was detected by annexin V labeled with FITC. Propidium iodide (PI) was used to determine cell necrosis. After exposure to various experimental conditions, the cells were trypsinized and labeled with fluorochromes, and then, cytofluorometric analysis was performed with a FACScan (Becton Dickinson, NY, USA). For the TUNEL assays, different groups of cells were grown on a coverslip, incubated with a TUNEL reaction mixture, counterstained with DAPI, and observed under a fluorescence microscope.

### 2.7. Western Blot Analyses

HDPCs were collected and lysed in the cell lysis buffer. Proteins were separated by electrophoresis and transferred onto a polyvinylidene difluoride (PVDF) membrane. Proteins bound by primary antibodies were visualized with an appropriate secondary antibody. The protein bands were detected using the Bio-Rad imaging system (Bio-Rad, Hercules, CA, USA) and quantified using NIH ImageJ software.

### 2.8. Intercellular ATP Level Determination

The ATP levels were detected by an ATP detection kit according to the manufacturer's instructions. The data was measured via a luminescence plate reader.

### 2.9. Functional Imaging Assays

After treatment with the indicated reagents, the cells were incubated with MitoSOX (2.5 *μ*M) or TMRM (100 nM). Images were captured under the fluorescence microscope. The NIH ImageJ software was used to measure and quantify the fluorescence signals. More than 100 clearly identifiable mitochondria in 10-15 randomly selected cells per experiment were measured in three independent experiments [17, 18].

### 2.10. Detection of Ca^2+^ Level

The cells were treated with the indicated reagents. Then, the cells were incubated with 10 *μ*M Fluo-4-AM (Beyotime, Shanghai, China), a Ca^2+^-sensitive fluorescent probe. The Ca^2+^ level was detected under a fluorescence microscope.

### 2.11. Data Analysis

Data were presented as the mean of three independent replicates ± standard deviation (SD) and considered significant at *p* < 0.05. Statistical analysis was performed using StatView software (version 5.0.1, SAS Institute, USA). For comparisons between the multiple groups, one-way ANOVA was used followed by individual post hoc Fisher tests when applicable.

## 3. Results

### 3.1. H_2_O_2_ Induced Cell Death and Mitochondrial Dysfunction in HDPCs

The cells around the pulp tissue, which showed a spindle shape and fibroblast-like morphology after being cultured for 5 days (Supplementary Figure 1a), were characterized as primary HDPCs. In addition, the cells exerted strong character of proliferation (Supplementary Figure 1b) and showed mesenchymal origin by the positive expression of vimentin in the cytoplasm but were negative for keratin (Supplementary Figures 1c and 1d).

**Figure 1:**
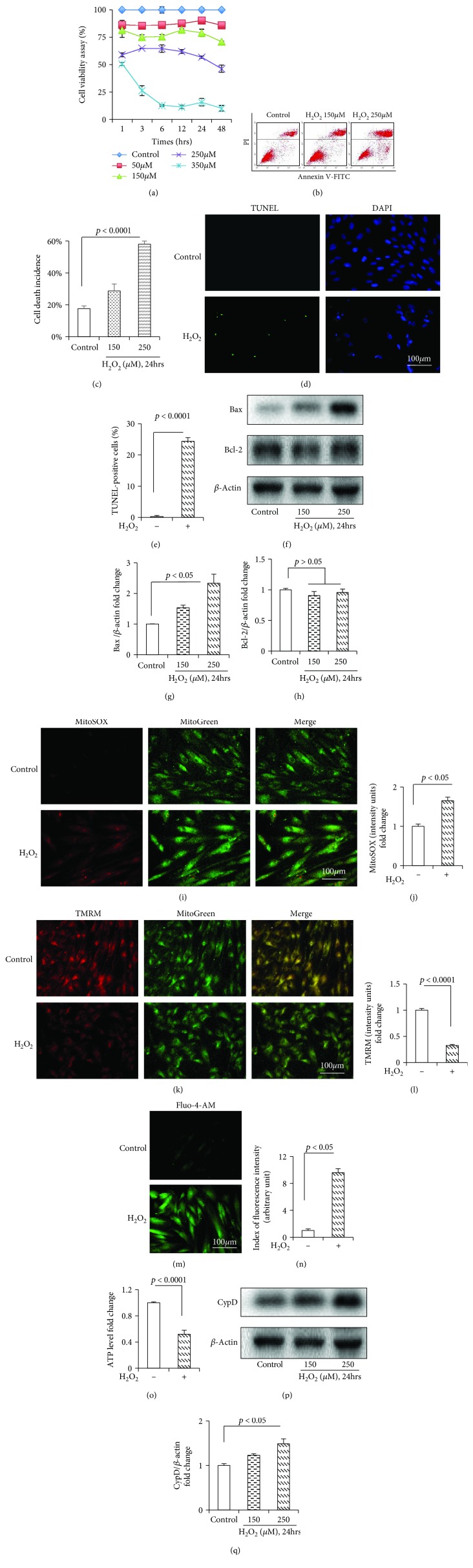
H_2_O_2_-induced cell death and mitochondrial dysfunction in the HDPCs. (a) Cell viability was determined by MTT reduction in the HDPCs with or without the presence of H_2_O_2_. (b, c) Flow cytometric quantification of cell death. HDPCs were treated for 24 h with culture medium or H_2_O_2_ (150 *μ*M, 250 *μ*M). (d, e) TUNEL staining and assay. (f) Representative immunoreactive bands with different densities for Bcl-2 and Bax in the HDPCs in the presence of H_2_O_2_. Quantification of immunoreactive bands for Bax (g) and Bcl-2 (h) relative to *β*-actin. Representative images showing MitoSOX staining (i) and quantification (j) in the indicated groups. Representative images with TMRM staining (k) and quantification (l) in the indicated groups. Representative images showing Fluo-4-AM staining (m) and quantification (n) in the indicated groups. ATP (o) in the indicated groups. (p) Densitometry of immunoreactive bands for CypD in the HDPCs in the presence of H_2_O_2_. (q) Quantification of immunoreactive bands for CypD relative to *β*-actin. HDPCs were treated for 24 h with H_2_O_2_ (250 *μ*M) (+) or culture medium (-). Data represent the mean values ± SD of three independent experiments.

As shown in Figure 1(a), H_2_O_2_ reduced HDPC viability in a time- and dose-dependent manner. The annexin/PI staining assay revealed a significant enhancement in the incidence of cell death. 250 *μ*M H_2_O_2_ markedly enhanced the rate of cell death compared with the control group (Figures 1(b) and 1(d)). The H_2_O_2_-induced DNA damage was further confirmed by the TUNEL staining (Figures 1(d) and 1(e)). Compared with the control group, H_2_O_2_ remarkably increased the protein level of proapoptotic Bax but did not influence antiapoptotic Bcl-2 (Figures 1(f) and 1(h)).

For the parameters of mitochondrial dysfunctions, H_2_O_2_ significantly increased the mitochondrial ROS (mtROS) level (MitoSOX staining) (Figures 1(i) and 1(j)) and decreased the mitochondrial membrane potential (MMP) (TMRM staining) (Figures 1(k) and 1(l)) in HDPCs. Furthermore, H_2_O_2_ markedly increased the intracellular Ca^2+^ level (Figures 1(m) and 1(n)) and reduced the ATP level (Figure 1(o)). In addition, H_2_O_2_ also significantly upregulated the expression of CypD (Figures 1(p) and 1(q)).

### 3.2. NAC Attenuated H_2_O_2_-Induced Cell Death and Mitochondrial Dysfunction in HDPCs

**Figure 2:**
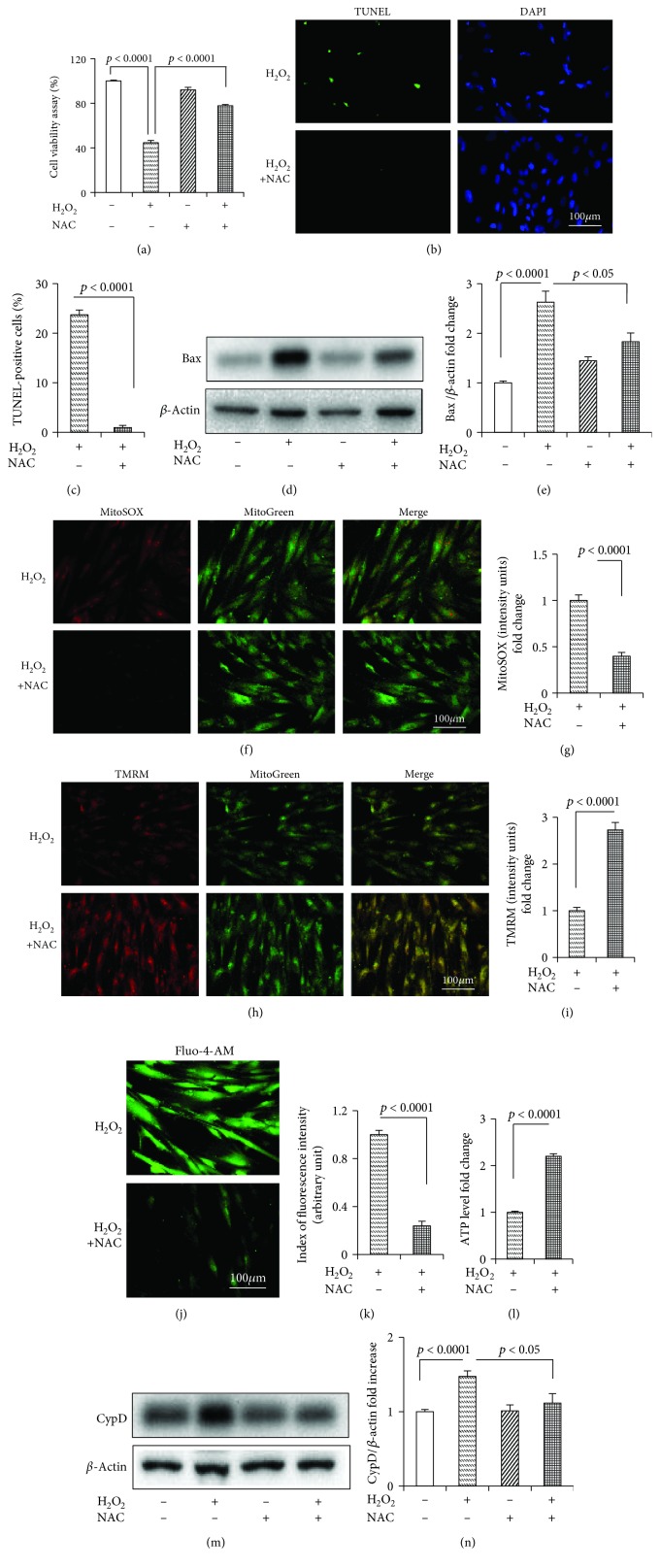
NAC attenuated H_2_O_2_-induced cell death and mitochondrial dysfunction in the HDPCs. (a) Cell viability determined by MTT reduction in the HDPCs in the presence of H_2_O_2_ with or without NAC. (b) TUNEL staining and (c) assay after NAC treatment. (d) Representative immunoreactive bands for Bax in the HDPCs with (+) or without (-) NAC treatment in the presence of H_2_O_2_ (+) or culture medium (-). Quantification of immunoreactive bands for Bax (e) relative to *β*-actin. Representative images showing MitoSOX staining (f) and quantification (g) in the indicated groups. Representative images showing TMRM staining (h) and quantification (i) in the indicated groups. Representative images showing Fluo-4-AM staining (j) and quantification (k) in the indicated groups. ATP (l) in the indicated groups. (m) Densitometry immunoreactive bands for CypD in the HDPCs with (+) or without (-) NAC treatment in the presence of H_2_O_2_ (+) or culture medium (-). (n) Quantification of immunoreactive bands for CypD relative to *β*-actin. HDPCs were treated for 24 h with (+) or without (−) NAC (2.5 mM) in the presence of H_2_O_2_ (250 *μ*M) (+) or culture medium (−). Data represent the mean values ± SD of three independent experiments.

NAC, a nonspecific antioxidant, observably preserved the cell viability in HDPCs treated with H_2_O_2_ (Figure 2(a)) and significantly mitigated cell death (TUNEL staining) (Figures 2(b) and 2(c)) and Bax expression (Figures 2(d) and 2(e)). NAC attenuated the effects of H_2_O_2_ by significantly suppressing mtROS (Figures 2(f) and 2(g)), increasing MMP (Figures 2(h) and 2(i)), decreasing the intracellular Ca^2+^ level (Figures 2(j) and 2(k)), and enhancing the ATP level (Figure 2(l)). NAC also decreased the CypD expression (Figures 2(m) and 2(n)).

### 3.3. Inhibition of CypD by CsA Reversed H_2_O_2_-Induced Cell Death and Mitochondrial Dysfunction in HDPCs

**Figure 3:**
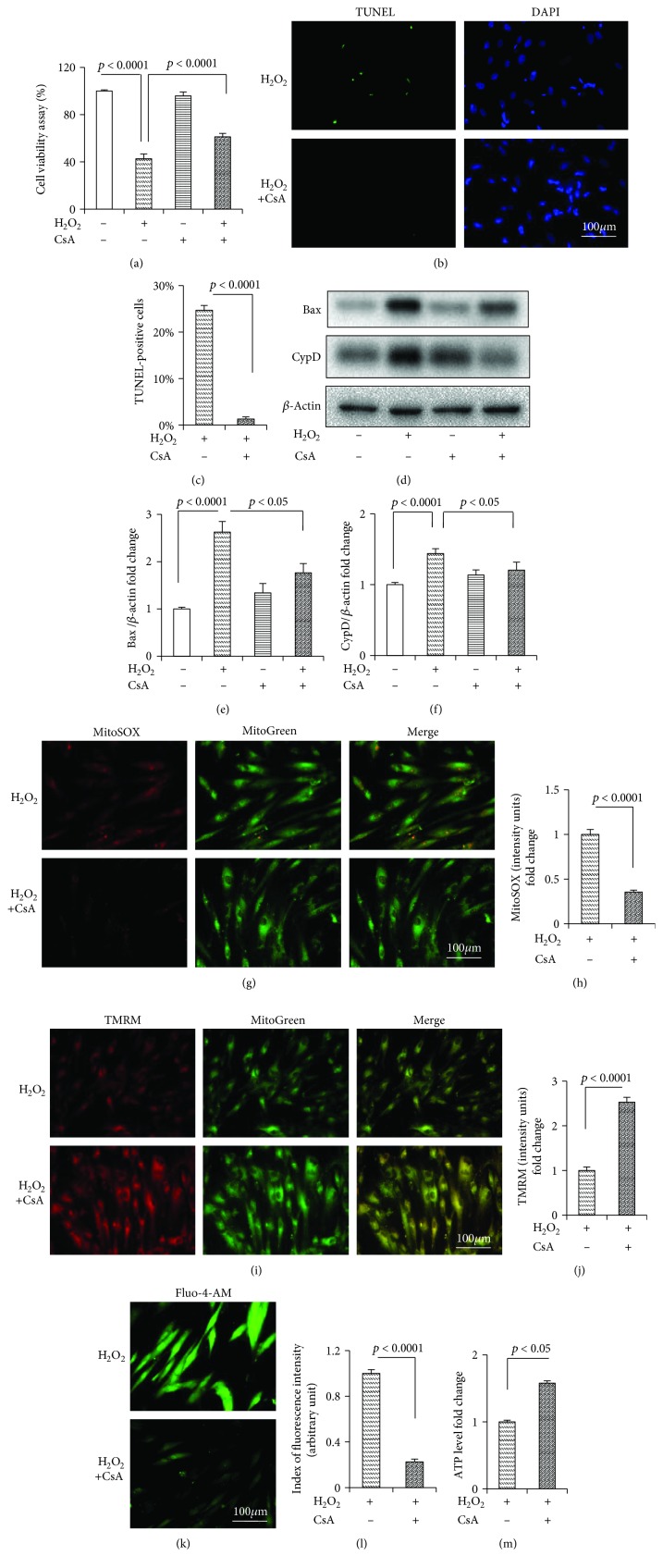
CsA attenuated H_2_O_2_-induced cell death and mitochondrial dysfunction in the HDPCs. (a) Cell viability determined by MTT reduction in the HDPCs in the presence of H_2_O_2_ with or without CsA. (b) TUNEL staining and (c) assay after CsA treatment. (d) Representative immunoreactive bands for Bax and CypD in the HDPCs with (+) or without (-) CsA treatment in the presence of H_2_O_2_ (+) or culture medium (-). Quantification of immunoreactive bands for Bax (e) and CypD (f) relative to *β*-actin. Representative images showing MitoSOX staining (g) and quantification (h) in the indicated groups. Representative images showing TMRM staining (i) and quantification (j) in the indicated groups. ATP (m) in the indicated groups. Representative images showing Fluo-4-AM staining (k) and quantification (l) in the indicated groups. HDPCs were treated for 24 h with (+) or without (−) CsA (2 *μ*M) in the presence of H_2_O_2_ (250 *μ*M) (+) or culture medium (−). Data represent the mean values ± SD of three independent experiments.

CsA, a pharmaceutical inhibitor of CypD, significantly inhibited the H_2_O_2_-induced cytotoxicity and death, as was evident by the MTT assay (Figure 3(a)) and TUNEL staining (Figures 3(b) and 3(c)), respectively. Furthermore, CsA significantly downregulated the expression level of Bax and CypD (Figures 3(d) and 3(f)) and abrogated mtROS (Figures 3(g) and 3(h)) and the intracellular Ca^2+^ level (Figures 3(k) and 3(l)). CsA also effectively increased the MMP (Figures 3(i) and 3(j)) and ATP level (Figure 3(m)).

### 3.4. Blockade of CypD by siRNA Mitigated H_2_O_2_-Induced Cell Death and Mitochondrial Dysfunction in HDPCs

**Figure 4:**
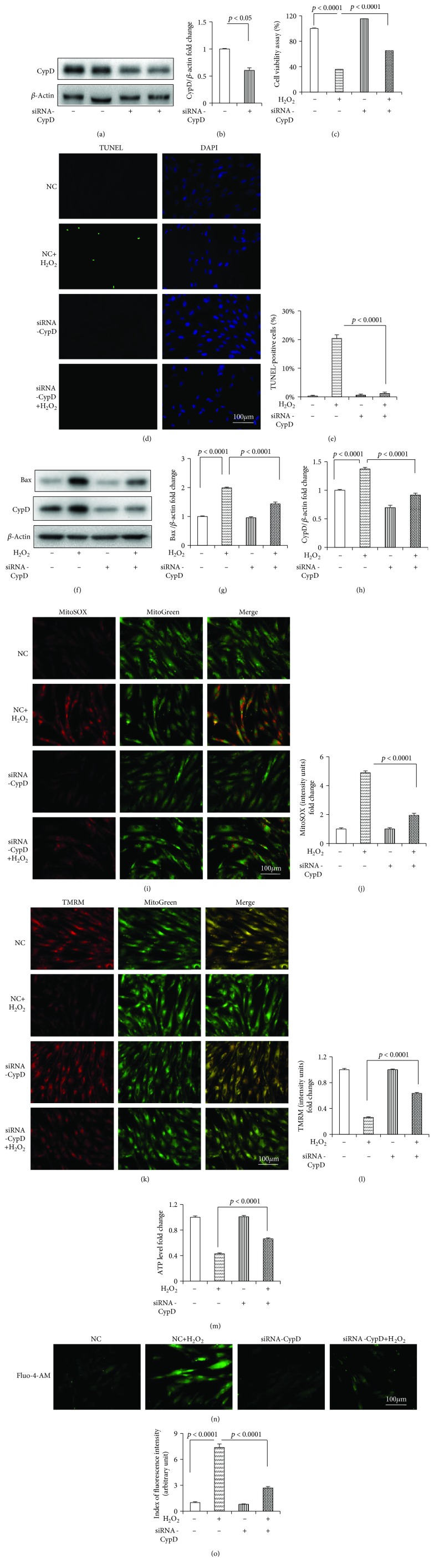
CypD siRNA attenuated H_2_O_2_-induced cell death and mitochondrial dysfunction in the HDPCs. (a) Representative immunoreactive bands for CypD in the HDPCs with CypD siRNA (+) or NC (-) treatment at a final concentration of 50 nM for 48 h. Quantification of immunoreactive bands for CypD (b) relative to *β*-actin. (c) Cell viability determined by MTT reduction in the HDPCs in the presence of H_2_O_2_ with or without siRNA-PPIF. (d) TUNEL staining and (e) assay after siRNA-PPIF treatment. (f) Representative immunoreactive bands for Bax, Bcl-2, and CypD in the HDPCs with (+) or without (-) siRNA-PPIF treatment in the presence of H_2_O_2_ (+) or culture medium (-). Quantification of immunoreactive bands for Bax (g), Bcl-2 (h), and CypD (i) relative to *β*-actin. Representative images showing MitoSOX staining (j) and quantification (k) in the indicated groups. Representative images showing TMRM staining (l) and quantification (m) in the indicated groups. ATP (n) in the indicated groups. Representative images showing Fluo-4-AM staining (o) and quantification (p) in the indicated groups. HDPCs were treated for 24 h with H_2_O_2_ (250 *μ*M) (+) or culture medium (−) in the CypD siRNA group (+) or the NC group (−). Data represent the mean values ± SD of three independent experiments.

The genetic knockdown of CypD with siRNA-PPIF was further conducted to validate the effect of CypD on HDPC death and mitochondrial dysfunction. Western blotting proved that siRNA-PPIF significantly downregulated the CypD expression (Figures 4(a) and 4(b)). We found that siRNA-PPIF significantly attenuated the H_2_O_2_-induced cytotoxicity (Figure 4(c)) and death (Figures 4(d) and 4(e)). siRNA-PPIF significantly inhibited the H_2_O_2_-induced expression of Bax and CypD (Figures 4(f) and 4(h)) and alleviated the mtROS and intracellular Ca^2+^ level (Figures 4(i), 4(j), 4(n), and 4(o)). siRNA-PPIF also protected the mitochondrial function from the adverse effect of H_2_O_2_ (Figures 4(k) and 4(m)).

## 4. Discussion

Substantial evidence has shown that many pathogenic stimuli can cause significant oxidative damage, leading to inflammation and necrosis in dental pulp tissues. However, hitherto, the underlying molecular mechanisms accounting for the OS-induced death of HDPCs remain elusive. In this study, we, for the first time, reported the key role of CypD in the OS-induced death of HDPCs. We found that pharmacological blockage and genetic reduction of CypD could significantly attenuate the oxidative damage to HDPCs. Our findings delineated novel insights into the crucial role of CypD-dependent mPTP opening and mitochondrial dysfunction in the OS-induced HDPC death.

Excessive ROS production can be induced in dental pulp by pathogenic stimuli, such as bacterial metabolite, dental bleaching, and unpolymerized resin monomers [2, 3, 19]. H_2_O_2_ is the most stable and commonly existing form of ROS and is widely used to establish cell OS injury models [20, 21]. In this study, we found that H_2_O_2_ significantly decreased the viability of HDPCs. Furthermore, H_2_O_2_ dramatically enhanced the death of HDPCs in comparison with the control group, as indicated by the results of flow cytometry and TUNEL assays.

Mitochondrial outer membrane permeation (MOMP) is a key event in apoptotic cell death, which is controlled by the Bcl-2 family proteins [22]. Bax and Bcl-2, Bcl-2 family proteins, are important in regulating apoptotic cell death. Bax is upregulated in response to DNA damage [23] and regulates mitochondria-dependent cell death [24]. In response to apoptotic stimuli, Bax oligomerizes and forms pores perforating the outer mitochondrial membrane [25, 26]. MOMP occurs during pore formation, and the size of the pore can vary according to the number of recruited Bax dimers [27]. Bcl-2 plays an important part in promoting cellular survival and bating the actions of proapoptotic proteins [28]. Bcl-2 antagonizes MOMP by blocking Bax oligomerization and pore-forming activity [29, 30]. Our immunoblot analyses showed that H_2_O_2_ significantly increased the expression of proapoptotic protein Bax in HDPCs, which was consistent with the previous findings from osteoblastic cells [18] and human fetal lung fibroblast cells [31]. On the other hand, different from these studies, we found that Bcl-2 was not significantly affected by H_2_O_2_, which might be due to the different cell types.

Increased ROS triggers cell death by inducing mitochondrial dysfunction [17, 32, 33]. NAC primarily abrogates mtROS and therefore mitigates myocardial cell death [20]. In the current study, H_2_O_2_ resulted in significant mitochondrial dysfunction, which was indicated by the increased mtROS, enhanced intracellular Ca^2+^ level, and decreased ATP level and MMP in HDPCs. NAC, in this study, not only blunted OS but also attenuated mitochondrial dysfunction in HDPCs. These results further confirmed that ROS-induced mitochondrial dysfunction played a key role in HDPC death. A mitochondria-specific antioxidant, mitoquinone, has been proved to be effective in ameliorating OS-related diseases [18, 34]. It would be highly interesting to apply these agents in this HDPC injury model to confirm the role of mitochondrial OS and dysfunction in OS-induced HDPC death in the future study.

The mPTP, initiated by severe OS and cytosolic Ca^2+^ overload, is a key regulator in cell death [35, 36]. The opening of mPTP leads to rapid loss of MMP (which abolishes all MMP-dependent mitochondrial activities, including ATP synthesis), osmotic breakdown of mitochondrial membranes, and structural breakdown of the organelle, eventually causing the regulated cell death [13, 35, 36]. In this study, OS was shown to lead to mPTP opening in HDPCs, as reflected by MMP dissipation and Ca^2+^ disorder, which would result in the increase in mtROS and energy failure (decrease in ATP). A similar pattern was also found in H_2_O_2_-treated RAW264.7 macrophages [37]. NAC successfully inhibited mPTP opening and rescued mitochondrial dysfunction, thereby attenuating HDPC death. Taken together, these results showed that (i) mPTP opening was implicated in HDPC death and (ii) OS was the major inducer of mPTP opening-induced death in the H_2_O_2_-treated HDPCs.

In our study, we mainly focused on the mPTP opening-induced cell death. Other than CypD, several proteins including the voltage-dependent anion channel (VDAC), adenine nucleotide translocator (ANT), and inorganic phosphate carrier (PHC) are supposed to be the putative modulators of mPTP [35]. However, previous studies using robust genetic tools confirm that CypD is the only vital protein in vivo required for mPTP opening [14, 38]. The knockout of VDAC, ANT, or PHC fails to protect cells from the mPTP opening [39–42]. In addition, mitochondrial F_1_F_O_ ATPase is also demonstrated to be involved in mPTP formation [43]. However, this conclusion is challenged by further reports by calculating their ion conductance and selectivity and especially the persistence of the mitochondrial permeability transition in the absence of the c-subunit of human F_1_F_O_ ATPase [44–46]. A very recent study largely gives structural explanation for the abovementioned inconsistent phenomena [47]. This study shows that there is no conventional mPTP formation but a CsA-sensitive channel when c-subunit is knocked out, still contributing to the depolarization of the inner mitochondrial membrane [47]. Further evidence will help to confirm the role of F_1_F_O_ ATPase in mPTP. In this study, we mainly focused on CypD and hypothesized that CypD was a key molecule in the H_2_O_2_-induced HDPC death. As expected, CsA and CypD siRNA inhibited the H_2_O_2_-induced mPTP opening, attenuated mitochondrial dysfunction, and prevented the death of HDPCs. Moreover, our results found that the CypD expression level significantly increased in the H_2_O_2_-treated HDPCs. This was consistent with a previous study showing that the increased expression of CypD contributed to the oxidative injuries in endothelial cells induced by a thyroid-stimulating hormone [16]. An *in vivo* study also confirmed that OS-induced cell death was mediated by CypD overexpression in the fibroblasts of patients with X-linked adrenoleukodystrophy [48]. Nonetheless, the overexpression of CypD was not always indispensable in its mediated cell death [49]. CypD may mediate the mPTP opening through three main mechanisms: (1) physical relationships with pore components [50], (2) ANT-independent pore formation [39], and (3) mitochondrial crista remodeling on the complete discharge of cytochrome C [51]. Consequently, CypD-mediated mPTP opening, especially in response to OS and Ca^2+^ overload, can be attenuated by either CypD genetic deletion [14], CypD peptidyl-prolyl *cis*- or *trans*-isomerase activity inhibition, or CsA molecular conformation [39]. All findings underscore a vital role of CypD-driven mPTP opening in OS-induced mitochondrial dysfunction and cell death of HDPCs. However, we only analyzed the role of CypD in H_2_O_2_-induced toxicity in dental pulp in vitro, and in vivo evidence should be achieved to corroborate the role of CypD.

The upstream molecules regulating CypD remain largely unknown in the H_2_O_2_-induced death of HDPCs. A previous study indicates that gallic acid potentiates the extracellular signal-regulated kinase (ERK) phosphorylation, leading to a decrease in CypD expression, which contributes to the neuroprotective effect on cerebral ischemia/reperfusion injury [52]. Some studies also show that an increased thyroid-stimulating hormone can activate CypD by inhibiting sirtuin-3 (SIRT3) in endothelial cells [16]. Therefore, the search for vital upstream regulators of CypD in HDPCs, such as ERK, SIRT3, or p53, needs to be further explored in the future study.

## 5. Conclusions

Our data offered new insights into the role of CypD-mediated mitochondrial dysfunction in the H_2_O_2_-induced HDPC death (Supplementary Figure 2) and the possible usage of the CypD inhibitor in the clinical treatment of dental pulp injury in the future.

## Data Availability

The data used to support the findings of this study are available from the corresponding authors upon request.

## Supplementary Materials

Supplementary Materials Figure S1: characterization of HDPCs. Figure S2: working hypothesis.
