# Supplementary material for: Blockade of Cyclophilin D Attenuates Oxidative Stress-Induced Cell Death in Human Dental Pulp Cells
Source: Oxid Med Cell Longev. 2019 Apr 4;2019:1729013. doi: 10.1155/2019/1729013 (PMC6476071; doi:10.1155/2019/1729013)
Supplement: Supplementary Materials — Figure S1: characterization of HDPCs. Figure S2: working hypothesis. [file 1729013.f1.docx]

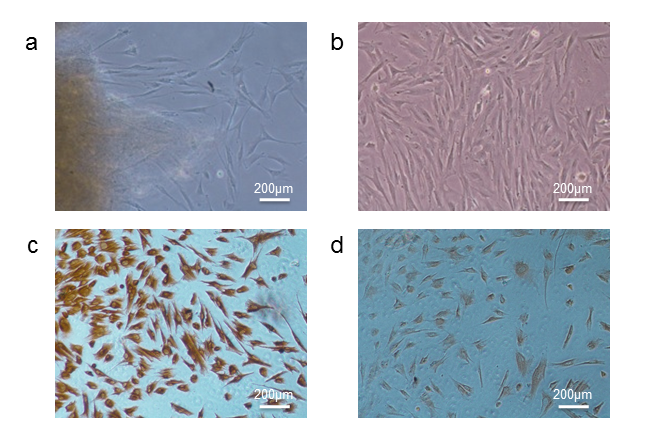


**Supplementary Figure 1.** Characterization of HDPCs. (a) There were spindle-shaped cells around human dental pulp tissue after 5 days of inoculation. (b) HDPCs in third-generation showed strong proliferation. Immunocytochemical staining of HDPCs showed positive for vimentin (c) but negative for keratin (d).


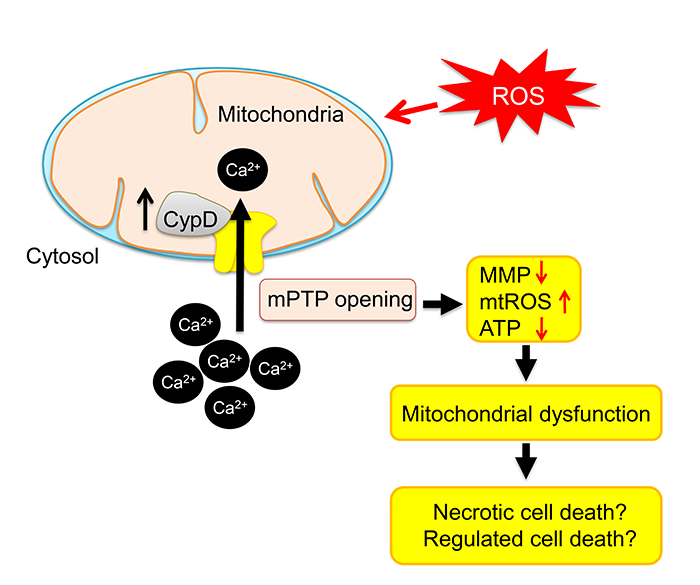


**Supplementary Figure 2.** Working hypothesis: CypD-dependent mitochondrial pathway in the OS-induced cell death in HDPCs
